# The Serotonin Transporter and Early Life Stress: Translational Perspectives

**DOI:** 10.3389/fncel.2017.00117

**Published:** 2017-04-26

**Authors:** Danielle J. Houwing, Bauke Buwalda, Eddy A. van der Zee, Sietse F. de Boer, Jocelien D. A. Olivier

**Affiliations:** ^1^Unit Behavioral Neuroscience, Department of Neurobiology, Groningen Institute for Evolutionary Life Sciences (GELIFES), University of GroningenGroningen, Netherlands; ^2^Unit Molecular Neurobiology, Department of Neurobiology, Groningen Institute for Evolutionary Life Sciences (GELIFES), University of GroningenGroningen, Netherlands

**Keywords:** serotonin transporter, early life, stress, gene × environment, 5-HTTLPR, human, rodent

## Abstract

The interaction between the serotonin transporter (SERT) linked polymorphic region (5-HTTLPR) and adverse early life stressing (ELS) events is associated with enhanced stress susceptibility and risk to develop mental disorders like major depression, anxiety, and aggressiveness. In particular, human short allele carriers are at increased risk. This 5-HTTLPR polymorphism is absent in the rodent SERT gene, but heterozygous SERT knockout rodents (SERT^+/−^) show several similarities to the human S-allele carrier, therefore creating an animal model of the human situation. Many rodent studies investigated ELS interactions in SERT knockout rodents combined with ELS. However, underlying neuromolecular mechanisms of the (mal)adaptive responses to adversity displayed by SERT rodents remain to be elucidated. Here, we provide a comprehensive review including studies describing mechanisms underlying *SERT* variation × ELS interactions in rodents. Alterations at the level of translation and transcription but also epigenetic alterations considerably contribute to underlying mechanisms of *SERT* variation × ELS interactions. In particular, SERT^+/−^ rodents exposed to adverse early rearing environment may be of high translational and predictive value to the more stress sensitive human short-allele carrier, considering the similarity in neurochemical alterations. Therefore, SERT^+/−^ rodents are highly relevant in research that aims to unravel the complex psychopathology of mental disorders. So far, most studies fail to show solid evidence for increased vulnerability to develop affective-like behavior after ELS in SERT^+/−^ rodents. Several reasons may underlie these failures, e.g., (1) stressors used might not be optimal or severe enough to induce maladaptations, (2) effects in females are not sufficiently studied, and (3) few studies include both behavioral manifestations and molecular correlates of ELS-induced effects in SERT^+/−^ rodents. Of course, one should not exclude the (although unlikely) possibility of SERT^+/−^ rodents not being sensitive to ELS. In conclusion, future studies addressing ELS-induced effects in the SERT^+/−^ rodents should extensively study both long-term behavioral and (epi)genetic aspects in both sexes. Finally, further research is warranted using more severe stressors in animal models. From there on, we should be able to draw solid conclusions whether the SERT^+/−^ exposed to ELS is a suitable translational animal model for studying 5-HTTLPR polymorphism and stress interactions.

## Introduction

Major depressive disorder, also known as major depression, carries the heaviest burden amongst all mental and behavioral disorders and is globally the largest contributor to years lived with disability (Ferrari et al., 2013). At any given time, over 4 percent of the global population suffers from major depression, with females being 1.7 times more likely than males to experience a depressive episode (Vos et al., 2012). Depression is diagnosed when signs and symptoms persist for at least 2 weeks and include daily feelings of depression, anxiousness or hopelessness and despair, insomnia, and anhedonia, i.e., loss of interest in activities that were once pleasurable (DSM-5; American Psychiatric Association, 2013). It has been widely acknowledged that both genetic and environmental factors contribute to the psychopathology of major depression, most likely by interacting in a complex and interdependent manner. For instance, aversive early life events such as childhood maltreatment contribute substantially to the risk of developing depression (Heim et al., 2004). However, the responsivity to such aversive early life events is significantly affected by the individual's genetic background.

A well-studied example of such gene × environment interactions is the influence of serotonin transporter gene (*SERT*, 5-HTT, or SLC6A4) variation on individual stress susceptibility (Caspi et al., 2003, 2010). The serotonin transporter is of major importance in regulating synaptic serotonin (5-HT) concentrations and signaling, and 5-HT synapses play a central role in the neural circuitry controlling mood and temperament. Disturbances in the serotonin system are known to contribute to the psychopathology of many psychiatric disorders (reviewed in Andrews et al., 2015). Various gene variants of SERT may interact to generate up to 20-fold differences in serotonin transporter gene expression and functional levels *in vitro* (Murphy et al., 2008). Of these SERT gene variants, the best-studied is the repeat length polymorphism in the promoter region of the serotonin transporter gene (5-HTTLPR). It is assumed that the human 5-HTTLPR drives allele-specific SERT promoter activity leading to differences in variations in transcriptional activity, and functional serotonin uptake. In the 5-HTTLPR, different lengths of the repetitive sequence containing GC-rich, 20–23-bp-long repeat elements in the upstream regulatory region of the gene have been identified. A deletion or insertion in the 5-HTTLPR are referred to as the 14-repeat short (S, low expressing) and the 16-repeat long (L, high expressing) alleles. However, 17 up to 22 repeat alleles (XL) also occur (Goldman et al., 2011). Furthermore, a single nucleotide polymorphism within or immediately outside the SERT gene results in two forms of the L-allele (L-A and L-G) and the S-allele (S-A and S-G) leading to transcriptional differences of the SERT gene. More specific, the L-G variant results in transcriptional activity similar to the short allele (Hu et al., 2006). Compared to the long variant, individuals carrying at least one allele with the short repeat in the promotor region have 60–70% lower SERT mRNA expression levels (Murphy et al., 2008; Wankerl et al., 2014) leading to reduced 5-HT uptake in lymphoblast cells (Lesch et al., 1996) and blood platelets (Greenberg et al., 1999; Nobile et al., 1999; Anderson et al., 2002). However, results on blood 5-HT levels are inconsistent. Whereas, an earlier study reported increased 5-HT levels in whole blood samples of patients with one (S/L) or two (L/L) long alleles (Hanna et al., 1998), other studies did not find differences in 5-HT storage of blood platelets (Greenberg et al., 1999; Anderson et al., 2002), or whole blood 5-HT levels (Betancur et al., 2002) among genotypes. Likewise, human studies on binding of SERT in both blood platelets and the brain show variable results, ranging from reduced SERT binding in S-allele carriers (Lesch et al., 1996; Stoltenberg et al., 2002) to no differences at all (Greenberg et al., 1999; Nobile et al., 1999; Anderson et al., 2002). Furthermore, cerebrospinal fluid levels of 5-HIAA, the main metabolite of 5-HT, was reported to be lower in healthy short allele carriers compared to long allele carriers (Williams et al., 2001). However, 5-HIAA cerebrospinal fluid levels did not differ between genotypes of healthy people (Jönsson et al., 1998) and depressed patients (Zalsman et al., 2006). Moreover, 5-HIAA cerebrospinal fluid levels seem to be dependent on gender and ethnicity (Williams et al., 2003). Remarkably, most studies only differentiate between the L and S variant and did not include XL alleles (17 up to 22 repeat alleles) or other allelic variants resulting from single nucleotide polymorphisms, possibly explaining the discrepancies in demonstrating a causal link between 5-HTTLPR genotype and *in vivo* SERT levels or function among the various studies. In addition, other variations such as in methodology, sampling size, or selection bias may have played an important role, but remain largely unclear.

At the behavioral level, human S-allele carriers show little evidence for changes in general behavioral functions compared to L-allele carriers. Large scale studies on neuroticism, a personality trait involved in the propensity to anxiety and depression, are inconsistent in finding an association with 5-HTTLPR variation (Sen et al., 2004; Terracciano et al., 2009). However, it was suggested that after a history of early life stress, such as childhood maltreatment, human S-allele carriers appear to be more stress sensitive, and are more likely to develop major depression (Caspi et al., 2003). Over the years, this link between the 5-HTTLPR genotype and depressive symptoms following stressful life events has been rather controversial. Multiple studies showing results against, but mostly in favor of this relationship have been performed (Munafò et al., 2009; Risch et al., 2009; Karg et al., 2011; McGuffin et al., 2011; Sharpley et al., 2014; Vrijsen et al., 2015). Given that about 70 percent of Caucasians have at least one copy of the short allele, the effects of these gene variants in the response to an environment with significant stressors may have a potential high-societal impact (Haberstick et al., 2015). The underlying mechanisms of these serotonin transporter gene variation × ELS interactions have gained increasing attention over the years as more mechanisms are being unraveled using animal models of SERT gene variation. In this review, we will (1) discuss the behavioral effects of ELS in heterozygous SERT knockout rodent and non-human primate models, (2) provide an overview of the underlying mechanisms by addressing (epi)genetic effects of ELS, lower SERT gene expression (heterozygous animals) and their interaction in the brain, (3) critically discuss the use of the heterozygous knockout rodent as a translational model to study early life stress induced psychopathology and (4) suggest relevant future studies. We selected literature focusing on both the SERT heterozygous genotype and some form of early life stress (we limitedly used literature about stressors during adulthood). Understanding the exact mechanisms underlying these interactions can be beneficial and is essential for the development of personalized treatment strategies in many mental disorders, including major depressive disorder.

## SERT gene variation and early life stress in rhesus macaques

**Figure 1:**
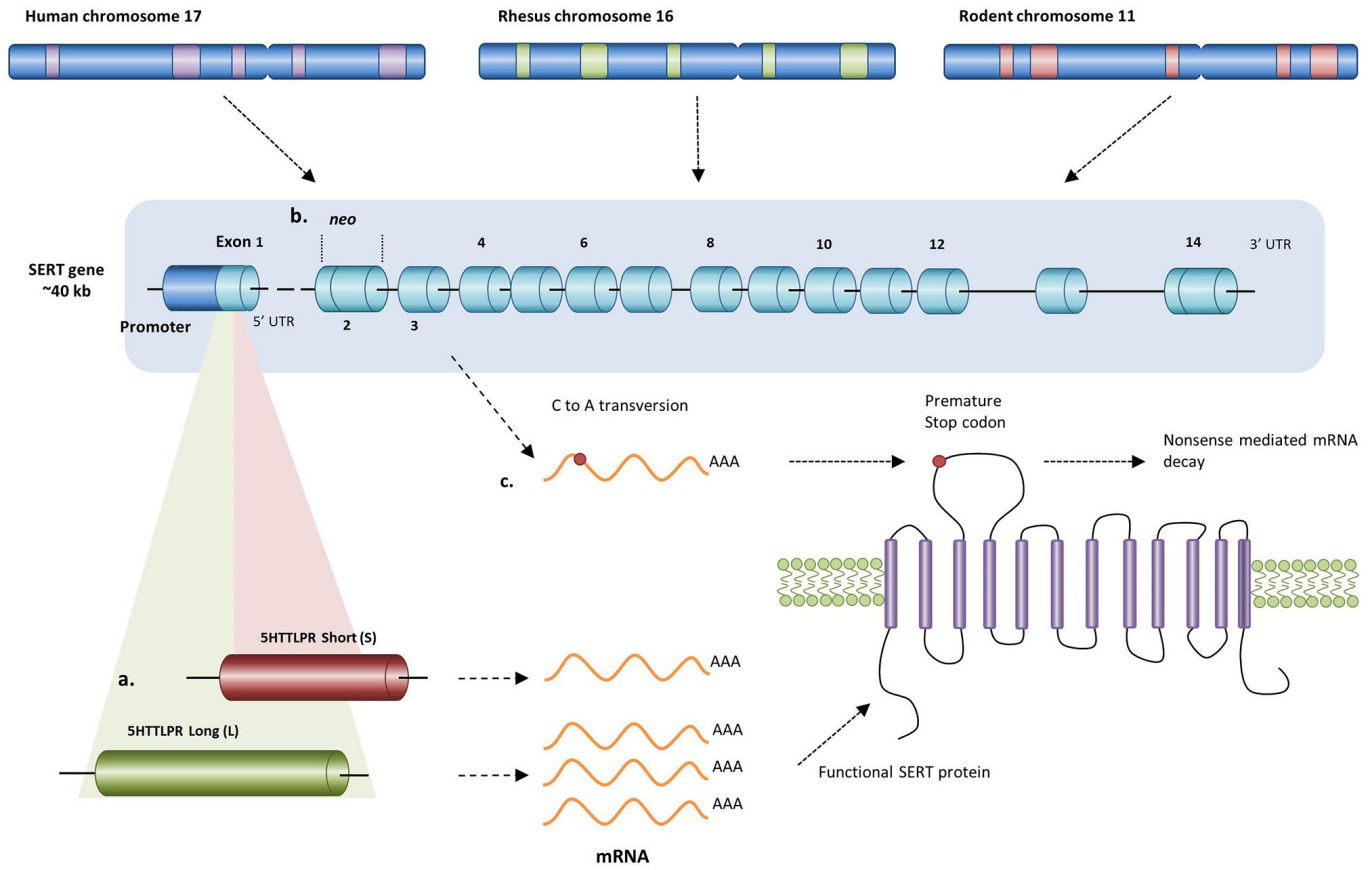
**Different alterations in the human, rhesus, and rodent SERT gene resulting in changed transcription levels of SERT. (a)** In humans and rhesus macaques having either a short or long allele for the 5-HTTLPR results in either lower or higher transcription levels respectively. Since rodents do not carry an orthologue of this polymorphism, knockout of the SERT can be achieved by **(b)** replacing exon 2 with a neo cassette (mice) or by **(c)** inducing a premature stop codon in exon 3 (rats) resulting in the absence of functional SERT protein.

Similar to humans, rhesus macaques carry an orthologue of the 5-HTTLPR, making it an excellent animal model to study the SERT gene polymorphism variants (Figure 1a; Lesch et al., 1997; Bennett et al., 2002). Promotor activity of the rhesus 5-HTTLPR long variant in an *in vitro* SERT promoter/luciferase gene construct, is almost twice as high as the short variant (Bennett et al., 2002). However, mRNA levels of SERT, hypothesized to be lowered as a result of lower transcriptional efficiency of the 5-HTTLPR short variant, were unaltered in peripheral blood cells compared to the long variant (Yu et al., 2010; Singh et al., 2012). Within the same genotype mRNA levels are highly variable indicating that other factors unrelated to the 5-HTTLPR genotype may influence SERT mRNA synthesis, stability, and degradation (Singh et al., 2012). Similar to human studies in lymphoblasts and blood platelets, 5-HT uptake in rhesus lymphocytes and peripheral blood cells (PBCs) is decreased in S-allele carriers (Singh et al., 2010, 2012). Furthermore, SERT binding was decreased and 5-HIAA concentrations were lower in peripheral blood cells of rhesus macaques carrying the short variant of the polymorphism (Singh et al., 2012). When taking into account the early rearing situation of the rhesus macaques, no differences in cerebrospinal fluid 5-HIAA levels were found among different genotypes of parent-reared rhesus macaques. However, when macaques were removed from their mother at birth and peer-reared, a form of early life social stress, cerebrospinal fluid 5-HIAA levels were lower in macaques with one short allele (S/L) compared to those with two long alleles (L/L) (Bennett et al., 2002). Furthermore, young rhesus monkey short allele carriers perform worse on orientation, attention and affective capacities but only in those macaques that were early deprived of their mother (Champoux et al., 2002). Peer-reared short allele carriers also display higher levels of aggression (Schwandt et al., 2010) and have increased sensitivity to alcohol consumption (Barr et al., 2003, 2004). Upon acute separation from either mother or peers, peer-reared S/L-allele carriers showed the highest levels of stereotypic behaviors compared to mother-reared S/L, mother-reared L/L, and peer-reared L/L carriers, whereas peer-reared L/L-allele carriers showed higher despair levels compared to mother-reared S/L, mother-reared L/L, and peer-reared S/L carriers (Spinelli et al., 2007). Overall, the rhesus 5-HTTLPR and early life stress interact to affect the behavioral outcome in rhesus macaques.

## Rodent models of SERT gene variation

Since rodents do not carry an orthologue of the human 5-HTTLPR, genetic variation in the human polymorphism can be simulated by creating serotonin transporter knockout (SERT^−/−^) rodents. For instance, using ENU-target driven mutagenesis, a premature stop codon has been induced in the SERT gene of rats resulting in a non-functional protein product (Figure 1c; Smits et al., 2004, 2006). In mice, a more common method is targeted disruption of the SERT gene by replacing exon 2 with a PGK-*neo* gene cassette using homologous recombination (Figure 1b; Bengel et al., 1998). Consequently, SERT knockout mice and rats, particularly the heterozygous ones, may demonstrate a similar loss-of-function in SERT activity as seen in the human and rhesus 5-HTTLPR genotype.

### Neurochemical differences

Heterozygous knockout animals (SERT^+/−^) show reduced SERT expression and function, as seen by 40–50% less SERT protein levels (Bengel et al., 1998; Homberg et al., 2007a), although levels may vary in different brain regions (Bartolomucci et al., 2010). With serotonin transporters being absent, it is expected that 5-HT uptake is limited, resulting in higher extracellular 5-HT levels, while tissue levels are expected to be decreased as shown in SERT^−/−^ rodents (Bengel et al., 1998; Fabre et al., 2000; Mathews et al., 2004; Shen et al., 2004; Kim et al., 2005; Homberg et al., 2007a,b; Fox et al., 2008; Olivier et al., 2008b). However, little neurochemical changes have been observed in the SERT^+/−^ rodent when compared to SERT^+/+^ animals (Table 1). SERT^+/−^ mice show similar basal extracellular 5-HT levels to SERT^+/+^ mice (Mathews et al., 2004; Kim et al., 2005). In SERT^+/−^ rats, basal extracellular levels have so far only been measured in the hippocampus, by our research group, and did not differ from SERT^+/+^ control rats (data not shown). In addition, intracellular 5-HT levels in SERT^+/−^ mice and rats appear unchanged compared to SERT^+/+^ rodents (Bengel et al., 1998; Tjurmina et al., 2002; Kim et al., 2005; Homberg et al., 2007a,b; Olivier et al., 2008a; Fox et al., 2008). Also, no differences in basal 5-HT tissue levels in the pituitary and the adrenal glands of SERT^+/−^ were observed compared to SERT^+/+^ mice (Tjurmina et al., 2002). Both SERT^+/+^ and SERT^+/−^ mice pituitary and adrenal gland 5-HT levels were elevated in response to immobilization stress (Tjurmina et al., 2002). Nonetheless, there are some neurochemical differences found between SERT^+/−^ and SERT^+/+^ rodents. Brain 5-HT turnover is lowered in the frontal cortex, hippocampus and striatum of SERT^+/−^ mice compared to SERT^+/+^ mice (Carola et al., 2008; Bartolomucci et al., 2010). In response to a stressor, SERT^+/−^ mice show an even further decrease in 5-HT turnover in the frontal cortex, while SERT^+/+^ mice do not (Bartolomucci et al., 2010). Also, there is a 13% decrease in 5-HT uptake in the hippocampus of SERT^+/−^ rats (Homberg et al., 2007a). Furthermore, 5-HT synthesis appears unaltered in most brain regions of SERT^+/−^ rats and mice when compared to SERT^+/+^ controls (Bengel et al., 1998; Kim et al., 2005; Homberg et al., 2007a; Fox et al., 2008). Only a reduction was found in the hypothalamus of SERT^+/−^ mice compared to SERT^+/+^ mice (Kim et al., 2005). Overall, neurochemical alterations in mice appear to be in agreement with findings in rats (see also Kalueff et al., 2010).

**Table 1:** **Basal intra- and extra-cellular 5-HT levels in SERT^+/−^ mice and rats vs. SERT^+/+^ controls**.

	**SERT^+/−^ rats**		**SERT^+/−^ mice**	
	**Intracell**.	**Extracell**.	**Intracell**.	**Extracell**.
Frontal cortex	→^4^^,^^8^	–	↑^2^→^6^^,^^1^	→^7^
Cerebral cortex	–	–	–	–
Prefrontal cortex Pituitary gland	→^5^	–	→^10^	–
Hypothalamus	–	–	→^3^^,^^6^	–
Raphe nuclei	–	–	–	–
Cerebrospinal fluid	→^5^	–	–	–
Striatum	–	–	→^2^^,^^3^^,^^6^	→^7^
Nucleus accumbens	–	–	–	–
Caudate putamen	→^4^	–	–	→^9^
Hippocampus	→^4^^,^^8^	–	→^2^^,^^3^^,^^6^	–
Brain stem	–	–	→^2^^,^^3^^,^^6^	–
Amygdala	→^4^	–	–	–

↑, increased; →, No difference; –, not determined.

1 , Bartolomucci et al. (2010);

2 , Bengel et al. (1998);

3 , Fox et al. (2008);

4 , Homberg et al. (2007a);

5 , Homberg et al. (2007b);

6 , Kim et al. (2005);

7 , Mathews et al. (2004);

8 , Olivier et al. (2008a);

9 , Shen et al. (2004);

10 *, Tjurmina et al. (2002)*.

To summarize, SERT^+/−^ rodents partly lack SERT gene expression, resulting in certain changes in the serotonergic system. Furthermore, the presynaptic functioning of dopaminergic and noradrenergic systems under basal conditions is not affected (Homberg et al., 2007a; Bartolomucci et al., 2010). The SERT^+/−^ rodent can be of high translational value since SERT^+/−^ rodents have reduced SERT gene expression levels similar to human S-allele carriers (Bengel et al., 1998; Homberg et al., 2007a). Like most studies in human S-allele carriers, SERT^+/−^ rodents show no alterations in basal 5-HT levels (Bengel et al., 1998; Tjurmina et al., 2002; Mathews et al., 2004; Shen et al., 2004; Kim et al., 2005; Homberg et al., 2007a,b; Fox et al., 2008; Olivier et al., 2008a), but do show reduced 5-HT uptake (Homberg et al., 2007a), which was also observed in rhesus monkeys (Singh et al., 2010, 2012). With respect to the neurochemical similarities, SERT^+/−^ rodents might therefore be most translational to the human S-allele carrier when it comes to studying early life stress-induced psychopathology.

### Differences in the neuroendocrine system

Other differences between SERT^+/−^ rodents and their wildtype counterparts involve the neuroendocrine system, in particular the hypothalamic-pituitary-adrenal (HPA) axis. Across species, the HPA axis provides an appropriate response system to salient environmental challenges and opportunities (e.g., stressors). Stressful stimuli activate hypophysiotrophic neurons in the paraventricular nucleus (PVN) of the hypothalamus that secrete releasing hormones, such as corticotropin-releasing factor (CRF) and arginine vasopressin, into the portal circulation of the median eminence. The releasing hormones act on the anterior pituitary to promote the secretion of adrenocorticotropic hormone (ACTH) into the systemic blood circulation (Smith and Vale, 2006). Consequently, circulating ACTH acts on the adrenal cortex to initiate the synthesis and release of glucocorticoids [Cortisol in humans and corticosterone (CORT) in rodents]. Receptors for these steroids are expressed throughout the body and the brain and mediate the genomic and non-genomic actions of glucocorticoids. Genomic actions occur following binding to glucocorticoid receptors (GRs) and/or mineralocorticoid receptors (MRs) that then act as transcription factors to regulate long-latency and biologically long-acting changes in gene transcription (de Kloet et al., 1998). The MR has a high affinity for endogenous glucocorticoids and is extensively bound even during the circadian nadir of corticosteroid secretion. The GR has a lower affinity and is extensively bound only at relatively high levels of corticosteroids, such as those that occur during stress responses. The GR seems to be the primary mediator of delayed glucocorticoid inhibition of stress responses. By contrast, non-genomic effects occur within minutes of glucocorticoid release and involve actions at the target cell membrane. This non-genomic signaling accounts for the fast negative feedback-inhibition of the HPA axis.

Both basal and stress-induced levels of various key players of the HPA axis appear to vary among the different genotypes (Table 2). Among SERT^+/−^ and SERT^+/+^ rodents, it was found that basal fecal corticosterone (CORT) levels did not differ (Jansen et al., 2010; Bodden et al., 2015). In response to stress, fecal CORT levels increased, especially in SERT^+/−^ mice (Jansen et al., 2010). However, it was found that basal plasma CORT levels were not altered (Li et al., 1999) or were lower in SERT^+/−^ mice compared to SERT^+/+^ mice (van den Hove et al., 2011). Furthermore, plasma CORT levels in response to stress increased in SERT^+/−^ and SERT^+/+^ mice, with no genotype difference (Li et al., 1999; Tjurmina et al., 2002; Bartolomucci et al., 2010). Although no differences were found between genotypes in basal ACTH plasma and pituitary ACTH levels (Li et al., 1999; Tjurmina et al., 2002), plasma ACTH levels were elevated in both SERT^+/+^ and SERT^+/−^ mice after immobilization stress, without affecting pituitary ACTH levels (Tjurmina et al., 2002). The stress-induced response in plasma ACTH levels after a saline injection was higher in SERT^+/−^ mice compared to SERT^+/+^ mice, suggesting increased responsivity toward stressful stimuli (Li et al., 1999). In response to a CRF injection, which stimulates secretion of ACTH and in turn CORT release, plasma ACTH and CORT levels were increased in both SERT^+/+^ and SERT^+/−^ mice (Jiang et al., 2009). Basal CRF mRNA levels in the PVN, as well as CRF receptor 1 (R1) binding sites were significantly reduced in SERT^+/−^ mice compared to SERT^+/+^ mice (Jiang et al., 2009). Also, basal pituitary CRF R1 mRNA levels were not different among SERT^+/+^ and SERT^+/−^ mice, whereas elevated plus maze stress lowered CRF R1 mRNA levels significantly in SERT^+/−^ mice. Even so, SERT^+/−^ mice displayed lower CRF R1 mRNA levels than SERT^+/+^ mice under stressed conditions (Jiang et al., 2009). Regarding feedback regulation, it was found that nuclear GR protein levels in the hypothalamus were lower in SERT^+/−^ compared to SERT^+/+^ mice, while after elevated plus-maze stress these levels returned to normal (Jiang et al., 2009). The lower GR protein levels are most likely the result of a similar decrease in basal GR mRNA levels in the PVN, pituitary and adrenal cortex of SERT^+/−^ mice. Furthermore, elevated plus maze stress did not affect GR mRNA levels in the PVN, but reduced GR mRNA levels in the pituitary of SERT^+/+^ mice compared to non-stressed controls, but not in SERT^+/−^ mice. However, in the adrenal cortex GR mRNA levels were significantly reduced in both SERT^+/−^ and SERT^+/+^ mice after elevated plus-maze stress (Jiang et al., 2009). Overall, most studies show alterations in HPA axis response and its feedback regulation in heterozygous SERT knockout mice, suggesting increased sensitivity to stressors.

**Table 2:** **Alterations in key components of the HPA axis in heterozygous SERT knockout mice and rats**.

	**Basal**		**Stress-induced**		**ELS-induced**		SERT × ELS interaction?^*^
	**SERT^+/+^**	**SERT^+/−^**	**SERT^+/+^**	**SERT^+/−^**	**SERT^+/+^**	**SERT^+/−^**	
**PARAVENTRICULAR NUCLEUS (PVN)**							
CRF mRNA	C	→^3^^,^^4^ ↓^7^	→^7^	↑^7^	–	→^3^^,^^4^	No^3^^,^^4^
MR mRNA	C	→^3^	–	–	–	→^3^	No^3^
GR mRNA	C	→^3^ ↓^7^	→^7^	→^7^	–	–	–
GR protein	C	↓^7^	→^7^	↑^7^	–	–	–
FKBP5 mRNA	C	→^3^	–	–	–	→^3^	No^3^
**PITUITARY**							
CRF R1 binding	C	↓^7^	↑^7^	↑^7^	–	–	–
CRF R1 mRNA	C	→^3^^,^^7^	→^7^	↓^7^	→^3^	→^3^	No^3^
MR mRNA	C	→^3^	–	–	→^3^	→^3^	No^3^
GR mRNA	C	→^3^↓^7^	↓^7^	→^7^	→^3^	→^3^	No^3^
FKBP5 mRNA	C	→^3^	–	–	↓^3^	→^3^	No^3^
Pro-opiomelanocortin mRNA	C	→^3^	–	–	→^3^	→^3^	No^3^
Plasma ACTH	C	→^3^^,^^9^^,^^8^	↑^8^^,^^9^	↑^9^^,^^8^	→^3^	→^3^	No^3^
Pituitary ACTH	C	→^9^	→^9^	→^9^	–	–	–
ACTH response to CRF	↑^7^	↑^7^	–	–	–	–	–
**ADRENAL CORTEX**							
Fecal CORT	C	→^2^^,^^6^	→^6^	↑^6^	–	–	–
Plasma CORT	C	↓^5^→^1^^,^^3^^,^^9^^,^^8^	↑^1^^,^^9^^,^^8^	↑^1^^,^^9^^,^^8^	→^3^	→^3^	Yes^3^
Adrenal CORT	C	→^9^	↑^9^	↑^9^	–	–	–
ACTH receptro mRNA	C	→^3^	–	–	↑^3^	→^3^	Yes^3^
GR mRNA	C	↓^7^	↓^7^	↓^7^	–	–	–
11β-hydroxylase mRNA	C	→^3^	–	–	↑^3^	→^3^	Yes^3^
steroidogenic acute regulatory protein mRNA	C	→^3^	–	–	→^3^	→^3^	Yes^3^
3βHSD1 mRNA	C	→^3^	–	–	→^3^	→^3^	Yes^3^
Tyrosine hydroxylase mRNA	C	→^3^	–	–	→^3^	→^3^	No^3^

*C, Control; ↑, increased; ↓, decreased; →, No difference; –, not determined. The basal level (non-stressed) of SERT^+/−^ rodents were compared with the basal level of SERT^+/+^ rodents (C). The data from stressed groups were compared with the basal levels in same genotype animals*.

* , these studies all included SERT^−/−^ rats to determine an interaction effect.

1 , Bartolomucci et al. (2010);

2 , Bodden et al. (2015);

3 , van der Doelen et al. (2014a);

4 , van der Doelen et al. (2015);

5 , van den Hove et al. (2011);

6 , Jansen et al. (2010);

7 , Jiang et al. (2009);

8 , Li et al. (1999);

9 *, Tjurmina et al. (2002)*.

### Behavioral differences

Altered brain 5-HT levels are associated with substantial changes in rodent behavior, in particular in homozygous SERT^−/−^ rodents (Kalueff et al., 2010). However, basal behavioral changes in SERT^+/−^ rodents are less apparent. Changes in baseline locomotor activity, anxiety-like behavior, aggression and sexual behavior among SERT^+/−^ vs. wild-type control rodents are often not observed (Holmes et al., 2003; Homberg et al., 2007b; Chan et al., 2011). On the other hand, SERT^+/−^ rodents show enhanced reversal learning and impaired object recognition after 8 h (Olivier et al., 2009; Brigman et al., 2010). In response to a stressor such as an inescapable foot shocks male SERT^+/−^ mice, but not rats (van der Doelen et al., 2013) showed increased helplessness compared to SERT^+/+^ mice, indicated by a higher escape latency (Muller et al., 2011) or less escapes (Pryce et al., 2012) during the test session where they could actually escape the foot shock. However, basal behavioral differences between SERT^+/−^ and SERT^+/+^ mice were not observed in fear conditioning or in anxiety and depressive-like behavior, including the open field, novelty suppressed feeding, and forced swim test (Muller et al., 2011).

Despite the significant reductions in serotonin transporter expression in SERT^+/−^ rodents as well as in humans carrying the 5-HTTLPR S-allele, basal behavioral consequences seem limited. Large scale studies on neuroticism related traits, including anxiety and depression, found no association with 5-HTTLPR variation. However, after a history of early life stress such as childhood maltreatment human short allele carriers are reported to be more prone to develop depressive disorders (Caspi et al., 2003). Therefore, using early life stress in SERT^+/−^ rodents could be of translational value to the human situation. SERT^+/−^ rodents can be used to elucidate the exact underlying neural and molecular mechanisms of gene × environment interactions, in particular SERT genotype × early life stress.

## Behavioral adaptations due to early life stress in rodent models of SERT gene variation

Animal models of early life adverse experiences consist of exposing animals to stressful procedures either pre- or post-natally, leading to considerable changes in behavioral and physiological responding of the offspring at later stages in life. Prenatal stress exposure, which is usually performed during the last week of pregnancy (corresponding to the late second trimester in human pregnancy; Homberg et al., 2010), can affect the development of offspring by transferring stress mediators from the pregnant mother to the fetus. Most likely this occurs by transport of maternal stress hormones across the placenta and induced release of placental hormones into the fetal blood circulation (Huizink et al., 2004). Postnatal stress, on the other hand, usually interferes with mother-pup interactions, which are essential for optimal brain development. At birth, the rat pup brain is not fully matured and continuous to develop. Approximately, postnatal days 1–10 of rat brain neurodevelopment are equal to the third trimester in humans (Dobbing and Sands, 1979; Andrews and Fitzgerald, 1997), and maturation of the rat cerebral cortex at postnatal day 12 and 13 is equivalent to that of the human neocortex at birth (Homberg et al., 2010). Disruption of mother-pup interactions can affect hypothalamic-pituitary-adrenal (HPA) axis regulation which may lead to considerable changes in neurobiology and behavior that persist into adulthood (Lajud and Torner, 2015). Interactions between SERT genotype and prenatal stress have so far only been studied in SERT knockout mice (van den Hove et al., 2011). A common method used as prenatal stress in mice is the maternal restraint-stress paradigm, which usually consists of placing individual pregnant dams in a small transparent cylindrical restrainer for multiple times per day. A study using this prenatal stress paradigm found that SERT^+/−^ mouse offspring had intact memory function in the novel object recognition test after a 3-h retention interval, while SERT^+/+^ mice showed impaired memory performance (van den Hove et al., 2011). In addition, prenatally stressed SERT^+/−^ mouse offspring appeared less anxious in the elevated zero-maze compared to SERT^+/+^ offspring. Interestingly, under control conditions both male and female SERT^+/−^ offspring show increased mobility compared to SERT^+/+^ mice, whereas only prenatally stressed female SERT^+/−^ offspring tended to show lower mobility in the forced swim test compared to non-stressed controls (van den Hove et al., 2011).

Other studies choose to use both pre- and postnatal stressors (Heiming, 2009; Heiming et al., 2011; Bodden et al., 2015). In particular, exposing pregnant and lactating mouse dams to bedding of unfamiliar male mice has been used as an early life stressor. Putting unfamiliar male bedding every 2–3 days in the home cage of the dams is claimed to simulate a threatening environment to the mother and her pups, as unfamiliar male olfactory cues can signal infanticide (Weber and Olsson, 2008). Mouse offspring growing up in this so-called dangerous environment showed overall increased levels of anxiety-like behavior in the light/dark box test, but this was not evident in the elevated plus-maze (Heiming, 2009). In addition, reduced locomotor activity was found in both the light/dark box test and in the open field. However, SERT^+/−^ mice behavior was not different from SERT^+/+^ in these behavioral measures. Since there was habituation of the stress response to the treatment procedure, it was suggested that the observed effects in the offspring result from a change in the amount of maternal care in response to the stressor. The maternal care provided is a crucial factor influencing offspring development. Indeed, in a follow-up study from the same authors it was shown that these mothers living in dangerous environments had increased levels of fecal corticosterone metabolites and displayed a decrease in various maternal care behaviors (Heiming et al., 2011). Surprisingly, in this study no clear effects of either housing condition or SERT genotype on offspring behavior were found. A subsequent study by these authors again used unfamiliar male bedding as an aversive stimulus, but only during the postnatal period (Kloke et al., 2013). In addition to control mouse dams that were given neutral bedding, they also studied communal nesting. This is considered a positive environment as pups growing up in a communal nest experience increased levels of maternal care and engage in higher levels of peer interactions (Branchi et al., 2013). SERT^+/−^ offspring showed no differences in anxiety-like and explorative behavior when compared to SERT^+/+^ offspring. Furthermore, no effects of housing condition on anxiety were observed in mouse offspring during various behavioral tests (Kloke et al., 2013). Reasons for these discrepancies between results of the different studies remain unclear, but might be possibly due to age differences in the offspring between the studies. In addition, the authors suggested that there might have been an unknown extra stressor in the first study, as the mortality rate in mothers was high. Also, in the last study, the stressor was only given postnatal, compared to both pre- and postnatal in the previous studies, which shows the importance of the developmental period during which a stressor is given. Regardless of genotype, when the offspring was given the opportunity to freely choose between novelty and familiarity during free exploration, offspring from the adverse environment tended to show reduced anxiety, while communal nesting tended to increase anxiety-like behavior in this test. These results were contradictory to the expected findings and the authors proposed that both completely safe and stable as well as a maternal environment with significant stressors could result in higher levels of maternal corticosterone levels and increased HPA axis reactivity and fearfulness in offspring. However, they did not measure corticosterone levels in the mothers. Minimal interaction effects between housing condition and genotype were found for anxiety-like behaviors.

The same early life stress paradigm of Heiming (2009) and Heiming et al. (2011) was used and extended by Bodden et al. (2015) who added social defeat as an additional stressor during adolescence in the mice that were pre-and postnatally exposed to unfamiliar male bedding (dangerous environment). Offspring from the neutral bedding group also received a receptive female mouse during adolescence as a beneficial stimulus (beneficial environment). During adulthood, offspring from both environments were either exposed to escapable social defeat or a receptive female mouse. In line with the previous studies, SERT^+/−^ offspring did not show altered anxiety-like and exploratory behavior compared to SERT^+/+^ mice. However, mice that grew up in the beneficial environment and were confronted with escapable social defeat in adulthood displayed overall lower levels of anxiety-like behavior compared to both groups of mice that grew up in a dangerous environment. No interaction effects were found between their life experiences and genotype.

In another study using a postnatal stressor mouse offspring received daily mild electric foot-shocks from postnatal day 7 through 13 (Carroll et al., 2007). Both before and after the stressor, SERT^+/−^, and SERT^+/+^ mice did not differ in anxiety-like behavior when subjected to the light/dark exploration test, elevated plus-maze, or open field. Also, no differences were found between these genotypes for depressive-like behavior during the second exposure to the forced swim test (Carroll et al., 2007). This indicates that SERT^+/−^ mice were not vulnerable to the effects of postnatal repeated foot-shock stress under the conditions met in this study. Possibly, repeated foot-shock is not lastingly acting as a stressor (Koolhaas et al., 2011) to resemble early life adversity.

Another postnatal stressor that is considered to be an aversive environmental stimulus is low maternal care. Genetically identical mice can differ in the amount of maternal care they give to their pups during development, either low or high as seen by differences in grooming and licking the pups (Carola et al., 2008). SERT^+/−^ offspring receiving low maternal care displayed enhanced anxiety-like behavior in the open field and elevated plus maze compared to high maternal care receivers, whereas SERT^+/+^ mice were not affected by this early life stressor. Furthermore, SERT^+/−^ mice receiving low maternal care showed increased depressive-like behavior in the tail suspension test as measured by latency to reach immobility (Carola et al., 2008).

Next to low maternal care, maternal separation is also considered to be an adverse early life event (Lajud and Torner, 2015), as pups are separated from their mother for multiple hours per day, usually during the first 2 weeks after birth. SERT^+/−^ rats that were separated for 3 h per day on postnatal day two to 14 showed lower escape latencies following an escapable foot-shock, compared to control SERT^+/−^ rats (van der Doelen et al., 2013). This early stress effect was not present in SERT^+/+^ rats. As SERT^+/−^ rats showed increased stress coping behavior after being exposed to maternal separation early in life, it was suggested that early life stress does not have to always result in negative consequences later in life, but may be a beneficial outcome to early life stress, especially when the stressor early in life was not too intensive (van der Doelen et al., 2013).

Overall, particularly after early life conditions that lower the level of maternal care, SERT^+/−^ mice and rats can be more sensitive compared to SERT^+/+^ mice and rats, to develop anxiety- and depressive-like behavior or increased stress coping (Carola et al., 2008; van den Hove et al., 2011; van der Doelen et al., 2013), whereas in other studies using different stressors no such interaction effects were found (e.g., Kloke et al., 2013; Bodden et al., 2015). Even so, the results so far do not show profound behavioral effects as a result of SERT^+/−^ genotype × ELS interaction.

## Mechanisms of SERT genotype × early life stress interactions

The observed behavioral manifestations in SERT^+/−^ rodents as a result of early life stress are assumed to be attributed to underlying molecular alterations throughout the body and brain. Most likely, one would expect to see adaptations at the level of serotonin homeostasis. Whereas, the normal stress response results in rapid changes in 5-HT levels and turnover (Tjurmina et al., 2002; Bartolomucci et al., 2010), early life stress did not induce long lasting alterations in 5-HT turnover (Carola et al., 2008). Nevertheless, maternal care level and 5-HT levels in the hippocampus interacted in 10-day old mouse pups. SERT^+/+^ and SERT^+/−^ mice experiencing low maternal care did not differ in their 5-HT levels, while SERT^+/−^ high maternal care receivers showed higher 5-HT levels and 5-HT turnover compared to SERT^+/+^ mice that received high maternal care (Carola et al., 2011). Besides the 5-HT homeostasis some other adaptations taking place at the stress regulating HPA axis and extra hypothalamic brain regions regulating HPA axis activity have been reported.

### The level of the HPA axis

One of the neuroendocrine systems through which the different SERT genotypes and early life stress interactions can be manifested is the HPA axis. In adult SERT^+/−^ rats, no alterations were found in plasma CORT and ACTH levels both basal (Li et al., 1999; Jiang et al., 2009) and after early life maternal separation stress (van der Doelen et al., 2014a). However, SERT^+/−^ mice showed higher plasma ACTH levels in response to a saline injection compared to SERT^+/+^ mice (Li et al., 1999), although conflicting results were found (Jiang et al., 2009). Furthermore, under basal conditions, CRF mRNA levels in the PVN of the hypothalamus of SERT^+/−^ mice were reduced relative to SERT^+/+^ mice (Jiang et al., 2009). Similarly, reduced glucocorticoid receptor (GR) mRNA levels were found in the PVN, pituitary and adrenal cortex of SERT^+/−^ mice (Jiang et al., 2009).

More effects of SERT genotype, early life stress and their possible interaction on gene expression levels of key HPA axis players have been assessed (See Table 2 for an overview). In the adrenal glands, interactions were found between genotype and early life stress (ELS) for ACTH receptor, 11β-hydroxylase (a mitochondrial enzyme responsible for the last step in glucocorticoid biosynthesis), steroidogenic acute regulatory protein (transports cholesterol into the mitochondria) and 3βHSD1 (involved in CORT synthesis) mRNA levels in rats (van der Doelen et al., 2014a). However, it should be mentioned that the authors included the SERT^−/−^ rats along with the SERT^+/−^ rats, interfering with the interaction data. When inspecting the data it seems that the SERT^−/−^ rats are mainly causing the interaction effects. Nevertheless, adrenal gene expression levels did not differ between SERT^+/+^ and SERT^+/−^ rats. More interestingly, postnatal maternal separation stress increased ACTH receptor and 11β-hydroxylase mRNA levels in SERT^+/+^ rats, while mRNA levels of SERT^+/−^ rats remained unaffected.

In the pituitary, chaperone FK506-binding protein 51 (FKBP5) mRNA levels were reduced in SERT^+/+^ but not in SERT^+/−^ rats after early life stress (van der Doelen et al., 2014a). Moreover, no effects of genotype or ELS on mRNA levels of pro-opiomelanocortin (a precursor protein of ACTH), glucocorticoid receptor (GR), and mineralocorticoid receptor (MR) were found in the pituitary of rats. Similarly, no significant effects of genotype or early life stress on mRNA levels were found for CRF, MR, GR, and chaperone FKBP5 in the PVN of the hypothalamus (van der Doelen et al., 2014a, 2015).

In summary, adaptations in the HPA axis response of SERT^+/−^ rodents are limited. Gene expression levels of all the assessed key HPA-axis players were not affected in SERT^+/−^ rodents after ELS. Only minor adaptations in plasma ACTH levels (after saline injection), together with some alterations in basal gene expression levels of CRF and GR in the PVN were found when comparing SERT^+/+^ with SERT^+/−^ animals (Jiang et al., 2009).

### Extra hypothalamic brain regions regulating HPA axis activity

Adaptations underlying genotype × early life stress interactions can also be present in extra-hypothalamic brain regions (see Table 3). In particular, the medial prefrontal cortex (mPFC), hippocampus and the amygdala are targeted by gluco- and mineralcorticoids and can indirectly regulate HPA axis activity. Early life maternal separation exposure affected GR mRNA levels in the dorsal mPFC and the dorsal hippocampus differently in SERT^+/+^ rats compared to SERT^+/−^ rats (van der Doelen et al., 2014b). ELS reduced GR mRNA levels in SERT^+/+^ rats, leading to lower GR expression in the dorsal mPFC compared to SERT^+/−^ rats that underwent ELS. Furthermore, in control animals and those exposed to ELS no differences in dorsal hippocampus, ventral mPFC, and central amygdala GR mRNA levels were found in SERT^+/−^ rats compared to SERT^+/+^ controls. In the ventral hippocampus and bed nucleus of the stria terminalis (BNST), ELS had a main effect on GR expression leading to an overall decrease in mRNA levels. Again, the authors took the SERT^−/−^ rats along in their analysis, making it difficult to separately interpret the data. However, no significant differences were found after *post hoc* tests between genotypes, therefore SERT^+/+^ and SERT^+/−^ rats probably do not have different GR expression in the ventral hippocampus after maternal separation. Furthermore, no effects of ELS, SERT genotype or their interaction were found for GR mRNA levels in the Edinger-Westphal nucleus in the midbrain (van der Doelen et al., 2017).

**Table 3:** **Overview of molecular adaptations to SERT gene variation, early life stress and their interaction**.

	**Basal**		**ELS-induced**		SERT × ELS interaction?^*^
	**SERT^+/+^**	**SERT^+/−^**	**SERT^+/+^**	**SERT^+/−^**	
**V. HIPPOCAMPUS**					
MR mRNA	C	→^4^	→^4^	↑^4^	Yes^4^^*^
GR mRNA	C	→^4^	→^4^	→^4^	No^4^^*^
FKBP5 mRNA	C	→^4^	→^4^	→^4^	No^4^^*^
BDNF mRNA	C	→^1^	↓^1^	↓^1^	No^1^^*^
**D. HIPPOCAMPUS**					
MR mRNA	C	→^4^	→^4^	↑^4^	Yes^4^^*^
GR mRNA	C	→^4^	→^4^	→^4^	Yes^4^^*^
FKBP5 mRNA	C	→^4^	→^4^	→^4^	No^4^^*^
BDNF mRNA	C	→^1^	→^1^	↑^1^	Yes^1^^*^
BDNF mRNA in CA1 region	C	→^2^^#^^,^^3^^#^	→^2^^#^↑^3^^#^	↑^2^^#^→^3^^#^	Yes^2^^#^No^3^^#^
**VENTROMEDIAL PFC**					
MR mRNA	C	→^4^	→^4^	→^4^	No^4^^*^
GR mRNA	C	→^4^	→^4^	→^4^	No^4^^*^
FKBP5 mRNA	C	→^4^	↓^4^	→^4^	Yes^4^^*^
BDNF mRNA	C	↓^1^	↓^1^	→^1^	No^1^
**DORSOMEDIAL PFC**					
MR mRNA	C	→^4^	→^4^	→^4^	No^4^^*^
GR mRNA	C	→^4^	↓^4^	→^4^	Yes^4^^*^
FKBP5 mRNA	C	→^4^	→^4^	→^4^	Yes^4^^*^
BDNF mRNA	C	→^1^	→^1^	↑^1^	Yes^1^
**ANTERODORSAL BNST**					
MR mRNA	C	→^4^	→^4^	→^4^	No^4^^*^
GR mRNA	C	→^4^	→^4^	→^4^	No^4^^*^
FKBP5 mRNA	C	→^4^	→^4^	→^4^	No^4^^*^
**OVAL SUB. BNST**					
CRF mRNA	C	→	→	→	No^5^^*^
**CENTRAL AMYGDALA**					
CRF mRNA	C	→^5^	→^5^	→^5^	No^5^^*^
MR mRNA	C	→^4^	→^4^	→^4^	No^4^^*^
GR mRNA	C	→^4^	→^4^	→^4^	No^4^^*^
FKBP5 mRNA	C	→^4^	→^4^	→^4^	No^4^^*^
BDNF mRNA	C	↓^2^^#^	→^2^^#^	→^2^^#^	No^2^^#^
**EDINGER-WESTPHAL NUCLEUS (MIDBRAIN**)					
CRF_1_R	C	→^6^	→^6^	→^6^	No^6^^*^
CRF_2_R	C	→^6^	→^6^	→^6^	No^6^^*^
GR	C	→^6^	→^6^	→^6^	No^6^^*^
Ucn1	C	→^6^	→^6^	→^6^	No^6^^*^
**SENSORY CORTEX**					
BDNF mRNA	C	→^3^^#^	↑^3^^#^	↑^3^^#^	No^3^^#^
**VISUAL CORTEX**					
BDNF mRNA	C	→^3^^#^	→^3^^#^	→^3^^#^	No^3^^#^

*C, Control; ↑, increased; ↓, decreased; →, No difference. The basal level (non-stressed) of SERT^+/−^ rodents were compared with the basal level of SERT^+/+^ rodents (C). The data from ELS groups were compared with the basal levels in same genotype animals*.

# *, Low vs. high maternal care in mice*.

* *, these studies all included SERT^−/−^ rats to determine an interaction effect*.

1 , Calabrese et al. (2015);

2 , Carola et al. (2008);

3 , Carola et al. (2011);

4 , van der Doelen et al. (2014b);

5 , van der Doelen et al. (2015);

6 *, van der Doelen et al. (2017)*.

Next to GR mRNA levels, MR mRNA levels were also assessed in SERT^+/+^ and SERT^+/−^ rats (van der Doelen et al., 2014b). In both the dorsal and ventral hippocampus MR mRNA levels were substantially increased after ELS in SERT^+/−^ rats, leading to higher levels compared to SERT^+/+^ animals that were exposed to ELS (van der Doelen et al., 2014b). Furthermore, an overall decrease in MR mRNA expression in the dorsal mPFC was found after ELS, but this analysis also included SERT^−/−^ rats. Nevertheless, SERT^+/+^ and SERT^+/−^ did not differ from non-stressed rats. Although, SERT^+/−^ rats did show significantly higher MR mRNA levels in the dorsal mPFC compared to SERT^+/+^ rats in both non maternally separated and separated animals (van der Doelen et al., 2014b). Despite the fact that SERT^−/−^ rats were taken along in the analysis, no effects of ELS, SERT genotype, or their interaction were found for MR mRNA levels in the ventral mPFC, BNST, and central amygdala (van der Doelen et al., 2014b).

Besides some alterations in GR and MR mRNA expression, FKBP5 mRNA levels in the dorsal and ventral mPFC were also differentially affected between genotypes (van der Doelen et al., 2014b). Again SERT^−/−^ rats were taken along in the interaction analysis making it difficult to separate the effects in SERT^+/−^ from SERT^+/+^ rats. Nevertheless, ELS exposure did not seem to affect the FKBP5 mRNA levels in the dorsal mPFC of both SERT^+/−^ and SERT^+/+^ rats. Furthermore, ELS decreased FKBP5 mRNA levels in the ventral mPFC of SERT^+/+^ rats, while no effect was found in SERT^+/−^ rats. Although no significant increase was found after ELS in SERT^+/−^ rats, FKBP5 mRNA levels were significantly higher compared to SERT^+/+^ rats. FKBP5 mRNA levels in SERT^+/−^ rats were not affected under control conditions. In the ventral hippocampus, FKBP5 expression was overall decreased after ELS, when including SERT^−/−^ rats. Again *post hoc* tests revealed no significant differences between SERT^+/+^ and SERT^+/−^ rats, therefore we assume these two genotype respond in a similar way to ELS concerning FKBP5 expression in the ventral hippocampus. FKBP5 mRNA levels in the dorsal mPFC, dorsal hippocampus, BNST, and central amygdala were unaffected by genotype, ELS or their interaction.

Furthermore, mRNA levels of the releasing hormone CRF in the Central amygdala and the BNST were not affected by ELS, SERT genotype or their interaction (van der Doelen et al., 2015). Urocortin 1 (Ucn1), belonging to the CRF family of proteins, complements the actions of CRF in the stress response regulation and is highly expressed in the Edinger-Westphal nucleus in the midbrain (Kozicz et al., 2011; Ryabinin et al., 2012) and interestingly these neurons do not habituate to chronic stress (Korosi et al., 2005). CRF and Ucn1 act through two G-protein coupled receptors, CRF R1 and R2. The mRNA levels of both Ucn1 and CRF R2 in the Edinger-Westphal nucleus were not affected by early life maternal separation or SERT genotype. SERT^+/−^ rats displayed similar levels compared with SERT^+/+^ rats (van der Doelen et al., 2017). In contrast, compared with SERT^+/+^ rats, CRF R1 mRNA levels were significantly decreased in SERT^+/−^ rats (van der Doelen et al., 2017).

In summary, again there are only some adaptations in gene expression levels of various HPA-axis regulators in extra hypothalamic brain regions as a result of ELS in SERT^+/−^ rodents. Only MR mRNA levels in the dorsal and ventral hippocampus, and CRF R1 mRNA levels in the Edinger-Westphal nucleus were affected. These effects might contribute to the underlying mechanisms of genotype × ELS interactions in rats, but they are highly brain region dependent making it difficult to establish the exact mechanism.

### Cellular and molecular level

As described in sections The Level of the HPA Axis and Extra Hypothalamic Brain Regions Regulating HPA Axis Activity, SERT genotype × ELS can result in adaptations at the HPA axis (van der Doelen et al., 2013, 2014a). Similar to Ucn1 mRNA levels, the number and density of Ucn1 neurons were also not affected by maternal separation, SERT genotype, or their interaction (van der Doelen et al., 2017). Furthermore, SERT^+/+^ and SERT^+/−^ rats do not differ in co-localization of Ucn1 neurons and 5-HT fibers or 5-HT innervation of the Edinger-Westphal nucleus (van der Doelen et al., 2017).

Other possible mechanisms of SERT genotype × ELS interactions involve alterations in expression of various genes other than those mentioned in previous sections. Expression of brain-derived neurotrophic factor (BDNF), which is important for normal development, survival, and plasticity of central nerve system neurons, is known to be altered in response to stress and is involved in the pathophysiology of psychiatric disorders (Angelucci et al., 2005; Duman and Monteggia, 2006). Under basal conditions, SERT^+/−^ rats showed lower total BDNF mRNA levels in the ventral mPFC compared to SERT^+/+^ animals. While early life stress decreased total BDNF mRNA levels in the ventral mPFC of SERT^+/+^ rats, early life stress did not alter total BDNF mRNA levels in SERT^+/−^ rats (Calabrese et al., 2015). Genotype effects were not seen in the dorsal mPFC, ventral and dorsal hippocampus (Calabrese et al., 2015). Also, a history of early life maternal separation stress resulted in a reduction of BDNF mRNA levels in the ventral hippocampus of SERT^+/−^ and SERT^+/+^ rats, but showed no differences between the genotypes (Calabrese et al., 2015). Without early life stress, BDNF mRNA levels in the dorsal part of the hippocampus were not affected by genotype. Even though BDNF mRNA levels are brain region specific, BDNF gene expression in the dorsal hippocampus and dorsal mPFC only increased in SERT^+/−^ rats after ELS and not in SERT^+/+^ rats (Calabrese et al., 2015). Similarly, BDNF mRNA levels in the CA1 region of the hippocampus were increased only in SERT^+/−^ mice that were exposed to low maternal care, and not in SERT^+/+^ mice (Carola et al., 2008). This ELS-induced increase in BDNF mRNA levels is already present early in development (Carola et al., 2011). In summary, early life stress modulates BDNF mRNA levels in a complex and area specific manner, with clear distinction between the ventral and dorsal areas of the hippocampus and prefrontal cortex. The reduced BDNF mRNA levels in response to ELS in the ventral hippocampus of SERT^+/−^ rats could contribute to the anxiety- and depressive-like phenotypes, while increased BDNF mRNA levels found in the dorsal hippocampus after ELS exposure may result in the beneficial effects on cognition, contributing to the “for better or worse” theory of Belsky.

Underlying mechanisms of SERT genotype × ELS interactions, more specifically prenatal stress, have also been studied in the hippocampus of female SERT^+/+^ and SERT^+/−^ mice (van den Hove et al., 2011). Micro-assay based expression profiling revealed that both SERT genotypes and prenatal stress exposure influenced hippocampal expression levels of genes involved in the mitogen-activated protein kinase signaling pathway and neurotrophin signaling. Mitogen-activated protein kinase signaling is known to play an important role in embryogenesis, cell differentiation, cell proliferation and cell death (Asaoka and Nishina, 2010; Keshet and Seger, 2010). In addition, gene expression patterns indicating that the regulatory effect of prenatal stress is dependent upon the SERT genotype, included genes affecting biological signaling pathways such as Wnt signaling, which is important in embryonic development and stem cell maintenance (van den Hove et al., 2011). Furthermore, expression levels of genes involved in cytokine-cytokine receptor interactions were affected (van den Hove et al., 2011). One possible mechanism contributing to the outcome in SERT^+/−^ rodents could be that SERT genotype × prenatal stress interaction leads to a disruption of the cytokine homeostasis eventually leading to enhanced vulnerability to stress (van den Hove et al., 2011).

### Epigenetic mechanisms

The above molecular adaptations undoubtedly are involved in the underlying mechanisms of SERT genotype × early life stress interactions, and may be the result of epigenetic changes. Epigenetic processes such as DNA methylation, post-translational histone modification or non-coding RNA are important candidates for regulating gene expression levels by inducing long-lasting changes in gene function, by activating and deactivating parts of the genome, without affecting the DNA sequence itself (Bird, 2007). DNA methylation occurs at cytosine-phosphate-guanine sites (Moore et al., 2013), which enables regulation of gene transcription and gene expression levels. DNA methylation is considered to be the most stable epigenetic modification, as changes in DNA methylation can be replicated through cell mitosis and may persist into adulthood (Dolinoy et al., 2006). Using the SERT genotype × prenatal stress paradigm, the role of DNA methylation in hippocampal gene expression levels of female SERT^+/+^ and SERT^+/−^ C57BL6/J mice and the link with the behavioral profile was studied (Schraut et al., 2014). SERT genotype, prenatal stress and their interaction affected expression levels of many genes in distinct ways and differentially affected DNA methylation of over 800 genes. In particular, the SERT genotype × prenatal stress interaction was associated with a differentially methylated genomic region of the myelin basic protein (Mbp) gene. The methylation status of two specific sites of the Mbp gene was linked to anxiety-related behavior and the expression of the Mbp gene was dependent on prenatal stress, the SERT genotype and its interaction. This strongly suggests that specific molecular mechanisms contribute to the behavioral outcome of the SERT genotype, prenatal stress and their interaction (Schraut et al., 2014).

Likewise, epigenetic mechanisms have been studied in maternally separated SERT knockout rats (van der Doelen et al., 2015, 2017). More specific, the effect of SERT genotype × early life maternal separation stress on DNA methylation of the corticotropin-releasing factor gene has been studied (van der Doelen et al., 2015). CRF neurons play an important role in the activity of the HPA axis and may therefore play an important role in the neurobiological mechanisms of depression (Smith and Vale, 2006). Both CRF mRNA levels and DNA methylation of the *Crf* promotor region in the central amygdala, the oval subdivision of the BNST and the PVN was not affected by ELS or different between SERT^+/+^ and SERT^+/−^ rats (van der Doelen et al., 2015). Also, DNA methylation of the *Ucn* promotor region in the Edinger-Westphal nucleus was not affected by genotype. On the other hand, ELS resulted in an increase in DNA methylation of cytosine-phosphate-guanine site 156 and cytosine-phosphate-guanine 49, although SERT^−/−^ rats were included in the analysis, the data without the SERT^−/−^ rats still imply in this direction (van der Doelen et al., 2017). However, *Ucn* promotor methylation differences did not result in altered Ucn protein levels, therefore further studies are needed to understand the functionality of DNA methylation of the *Ucn* promotor region (van der Doelen et al., 2017).

## Conclusions and future directions

In this review, an overview of SERT gene variation × early life stress interaction and the currently known underlying molecular mechanisms have been provided, specifically by comparing SERT^+/+^ with SERT^+/−^ animals. However, few early life stressors interact with SERT gene variants resulting in both behavioral and molecular alterations. Similar to human S-allele carriers, SERT^+/−^ rodents can display increased anxiety- and depressive-like behavior in response to early life stressors. Studies using unfamiliar bedding to induce low maternal care, or maternal restraint-stress as a prenatal stressor show little or no effects at all on anxiety levels in SERT^+/−^ mice (Heiming, 2009; Heiming et al., 2011; van den Hove et al., 2011; Kloke et al., 2013; Bodden et al., 2015). Similarly, daily foot-shocks on postnatal day 7–13 are not sufficient to elicit anxiety- and depressive-like behavior in SERT^+/−^ mice (Carroll et al., 2007). However, prenatal restraint-stress did increase depressive-like behavior in SERT^+/−^ offspring, but these mice were strikingly less depressive-like than SERT^+/+^ before ELS (van den Hove et al., 2011). The most profound and convincing behavioral effects were found when SERT^+/−^ mice were subjected to low maternal care early in life, resulting in increased anxiety and depressive-like behaviors (Carola et al., 2008). Even so, this early-life stress method always compares low vs. high maternal care, and does not include normal maternal care levels. Similar studies also including normal maternal care should investigate whether the observed interaction effects are still present.

Furthermore, early life stress can even have a positive outcome on rodent behavior. Stress in the form of chronic maternal separation resulted in increased stress coping behavior in SERT^+/−^ rats, as seen by shorter escape latencies after an escapable foots-hock (van der Doelen et al., 2013). Nonetheless, it was proposed that SERT^+/−^ animals are not simply more susceptible to adverse early life experiences, but also seem to be more sensitive for beneficial experiences. For instance, the positive experience of cohabitation with a female mouse reduced anxiety-like behavior in the open field in SERT^+/−^ but not in wildtype mice (Kästner et al., 2015). Similarly, human S-allele carriers are more sensitive to positive emotions of their partner (Schoebi et al., 2012) and have a strong bias toward both negative and positive affective pictures (Fox et al., 2011) compared to non S-allele carriers. This concept of “for better and worse” suggests that genes “sensitive to stress” actually are plasticity genes and that individuals vary in their plasticity to environmental conditions (Belsky et al., 2009). Furthermore, the outcome of SERT gene variation × early life stress interactions can be of adaptive value. In particular, when the early life environment matches the adult environment, the observed behavioral responses, such as stress coping, can have high adaptive value. According to the match-mismatch hypothesis, stress coping responses are adaptive when they match current stress conditions, but are maladaptive when the conditions mismatch (Schmidt, 2011).

Overall, there is potential that the SERT genotype can determine an animals' stress response to their environment. However, it remains to be established what underlying mechanisms regulate SERT variation × ELS interaction and in particular the corresponding behavioral manifestations. One of the mechanisms through which SERT variation × maternal separation can interact is by inducing adaptations in HPA axis regulation. Overall, it seems that stress can induce a stronger increase in plasma ACTH levels in SERT^+/−^ rodents than in controls suggesting increased stress sensitivity. Some other HPA axis regulators were also differently affected in SERT^+/−^ compared to SERT^+/+^ rodents in response to stress (Table 2). In humans HPA axis hyperactivity has been found in depressed patients, seen by increased salivary cortisol levels (Juruena et al., 2006; Cowen, 2010).

Furthermore, early life stress did not result in altered mRNA levels of the HPA axis key components or an interaction in SERT^+/−^ rodents. In the PVN, pituitary and adrenal cortex, only SERT^+/+^ rodents were in some cases affected by early life stress. Underlying mechanisms in areas that regulate the HPA axis, such as the PVN and pituitary have been most extensively studied in male rats that underwent the maternal separation stress for 3 h per day from postnatal day 1–14 (van der Doelen et al., 2014a,b, 2015, 2017; Calabrese et al., 2015). Hypothalamic gene expression levels of various key HPA axis activity components such as CRF, MR, GR, and FKBP5 were not affected by SERT genotype × ELS interactions (van der Doelen et al., 2014a). However, in various extra-hypothalamic brain regions SERT genotype and ELS did induce adaptations of MR, GR, and FKBP5 mRNA levels in a brain area-specific way (van der Doelen et al., 2014b). In humans, major depression has been associated with an imbalanced response of glucocorticoid receptors (de Kloet et al., 2007). However, the exact direction of altered glucocorticoid mRNA levels is not always similar between humans and rodents, and might depend on the specific brain region. For example, MR mRNA levels are similarly decreased in the anterior hippocampus of depressed patients (Medina et al., 2013) and in the corresponding ventral hippocampus of SERT^−/−^ rats (van der Doelen et al., 2014b). However, the interaction of SERT genotype and ELS resulted in increased MR mRNA levels in the ventral hippocampus of SERT^+/−^ rats (van der Doelen et al., 2014b). Furthermore, SERT variation × ELS interactions are associated with adaptations in gene expression levels of neurotrophic factor BDNF (Calabrese et al., 2015). Overall, SERT variation and maternal separation resulted in a decrease in ventral hippocampus BDNF mRNA levels, although in the ventromedial PFC BDNF mRNA levels were only lowered in the SERT^+/+^ and not in SERT^+/−^ rats. Interestingly, maternal separation stress resulted in increased BDNF mRNA levels in the dorsal hippocampus and dorsomedial PFC of the SERT^+/−^ rat (Calabrese et al., 2015). These increased *bdnf* expression levels might contribute to the observed enhanced stress coping behavior in SERT^+/−^ rats that have been exposed to maternal separation stress in an earlier study (van der Doelen et al., 2013). In addition, CRF mRNA levels negatively correlated with the escape latencies of these animals, thus providing a neural correlate for stress coping behavior (van der Doelen et al., 2015). Furthermore, SERT genotype and maternal separation stress interacted to alter DNA methylation of the *crf* promotor region in the central amygdala, but not CRF mRNA levels (van der Doelen et al., 2015). This suggests that additional mechanisms are involved in regulating *crf* expression.

Unfortunately, most of these studies combining SERT genotype and ELS only investigated mRNA levels of genes of interest, while not taking into account the protein levels. Protein levels do not always correlate with mRNA levels and thus protein levels should be verified (Vogel and Marcotte, 2013). In addition, most studies on ELS in SERT variants do not scrutinize the molecular effects at the level of serotonin homeostasis, while the serotonin transporter is obviously of great importance in SERT heterozygous knockout rodents. Also noteworthy, epigenetic repercussions of ELS in SERT heterozygous knockout rodents are currently limited and more studies investigating methylation of the serotonin system-related genes and HPA axis associated genes would be of significant importance. In humans, ELS in the form of childhood abuse is associated with increased methylation of the SERT promotor (Beach et al., 2010, 2011). In rhesus monkeys only a genotype effect was discovered for SERT promotor methylation, with S-allele carriers showing higher cytosine-phosphate-guanine methylation levels (Kinnally et al., 2010). In the van der Doelen study (2017) it was shown that a similar increase in DNA methylation patterns was found in the *Ucn* promoter of SERT^+/−^ rats compared to SERT^+/+^ when exposed to early life stress, however these increasing levels did not result in different protein expression levels, confirming again that mRNA or epigenetic alterations do not always result into altered protein levels. More research is necessary to understand the functionality of the epigenetic processes.

Importantly, not only early life stressors such as childhood maltreatment, but also stressful events later in life can interact with the 5-HTTLPR and increase the risk for developing depression (Caspi et al., 2003). Likewise, acute or chronic stress later in life can affect behavior of the SERT knockout rodents [reviewed in van den Hove et al. (2011) and Homberg, 2012]. For example, repeated social defeat results in increased social avoidance in adult SERT^+/−^ mice compared to SERT^+/+^ mice (Bartolomucci et al., 2010). Most rodent studies use environmental stressors during a single period in life and thus do not take into account possible interactions with the environment later in life. Therefore, using multiple stressors in both the early and adult life might enhance SERT genotype × stress interaction effects on brain and behavior. An interesting way to study these environmental influences both early and later in life is for example by using a semi-natural environment, where rodents are able to express their full repertoire of behaviors (Snoeren et al., 2015).

In addition, most studies investigating SERT variation × ELS interactions show differences in methodology. The timing of stressors (prenatal, postnatal), the number of stressors per day (how often, how long) as well as how many days the stressor is given differ among studies. Furthermore, different species (mouse or rat) or even different strains might result in discrepancies between studies. Another important notion is the fact that nearly all studies use only male rodents to minimize variation due to hormonal fluctuation. Human females are 1.7 times more likely to develop depression (Vos et al., 2012) and SERT variation × ELS interactions may differ between sexes. Therefore, further studies should include both males and females. Furthermore, most studies focusing on underlying molecular mechanisms came from the same research group using rat SERT variation × maternal separation stress. ELS-induced effects in extra hypothalamic brain regions regulating HPA axis activity are not supported by effects on depressive-like behavior and even show a positive effect for SERT^+/−^ rats compared to SERT^+/+^ rats (van der Doelen et al., 2014a, 2015). Three hours of maternal separation may be too mild to produce adverse long term behavioral alterations in both SERT^+/−^ and SERT^−/−^ rats, and in line with the Belsky theory, longer maternal separation periods each day and applied at irregular and thus more unpredictable intervals may be more effective. When maternal separation occurs at fixed time intervals mothers can anticipate the absence of their pups and provide extra maternal care before and after separation, preventing adverse long term behavioral effects in the offspring (Franklin et al., 2010). Indeed, unpredictable maternal separation can induce depressive-like behavior in adult mouse offspring across generations (Franklin et al., 2010).

In conclusion, the exact molecular mechanisms underlying *SERT* genotype × ELS interactions appear to be highly complex, and diverse molecular alterations at the level of translation and transcription are substantially contributing to it. ELS induced alterations in SERT^−/−^ rodents may be less suitable to do research on *SERT* mechanisms, since total absence of the SERT does not exist in humans, although it might generate possible ideas about the importance of the SERT and serotonin homeostasis in behavior. Nevertheless, SERT^+/−^ rodents might be more translational to study SERT genotype × ELS interactions. Exposing the heterozygous SERT knockout rodent to an adverse early rearing environment may be of high translational value to the more stress sensitive human S-allele carrier, considering the similarities in neurochemical alterations. So far, most studies fail to show solid evidence for increased vulnerability to develop anxiety or depressive-like behavior after ELS in heterozygous knockout rodents. In addition, most molecular findings are obtained in studies that do not confirm depressive-like behavioral manifestations. Hence, further research is warranted as current studies might be insufficient. Main reasons may be: (1) stressors used might not optimal to induce maladaptations (e.g., not severe enough); (2) effects in females are largely absent due to a lack of studies, (3) few studies include both behavioral outcomes and molecular correlates of ELS-induced effects in SERT knockout rodents. The question arises whether the heterozygous SERT^+/−^ rodent creates a serotonergic subfunctional model. Although it is generally assumed that the approximately 50% remaining SERT molecules lead to differences in various processes in an adult animal, the supporting data is suggesting the opposite. Serotonergic functioning (Table 1) looks comparable to wildtypes. To find out either of these possibilities it is essential to try to elucidate the underlying mechanisms of *SERT* × ELS interactions, in particular in the heterozygous SERT knockout rodent. By extensively studying and including both long term behavioral and (epi) genetic aspects in both sexes, underlying mechanisms can be directly correlated. Moreover, more severe and realistic stressors should be applied to increase the chance that a not-optimally functioning serotonergic system (the SERT^+/−^) might be influenced leading to lasting changes in stress sensitivity. The rodent SERT^+/−^ × ELS model has potentially high potential, but future research is indispensable before a solid conclusion can be drawn whether this is a suitable translational animal model for ELS-induced psychopathology in psychiatric disorders.

## Author contributions

Drafting and/or revising the paper, including final approval to publish: DH, BB, EvdZ, SdB, and JO.

## Funding

This work was financially supported by the Marie Sklodowska-Curie IF (grant SEP-210188707) and the Swedish Society of Medicine (grant SLS-411161).

### Conflict of interest statement

The authors declare that the research was conducted in the absence of any commercial or financial relationships that could be construed as a potential conflict of interest. The reviewer CP and handling Editor declared their shared affiliation, and the handling Editor states that the process nevertheless met the standards of a fair and objective review.

## Abbreviations

5-HT: serotonin
5-HTT: serotonin transporter
5-HTTLPR: repeat length polymorphism in the promoter region of the 5-HTT gene
ACTH: adrenocorticotropic hormone
BDNF: brain-derived neurotrophic factor
BNST: bed nucleus of the stria terminalis
CORT: corticosterone
CRF: corticotropin-releasing factor
CRF R1 or R2: corticotropin-releasing factor receptor 1 or 2
ELS: early life stress
FKBP5: chaperone FK506-binding protein 51
GR: glucocorticoid receptors
HPA: hypothalamic-pituitary-adrenal
mPFC: medial prefrontal cortex
MR: mineralocorticoid receptors
PVN: paraventricular nucleus
SERT: serotonin transporter
SERT^+/+^: wildtype for the serotonin transporter
SERT^+/−^: heterozygous for the serotonin transporter
SERT^−/−^: knockout for the serotonin transporter
Ucn1: Urocortin 1
